# Fluorescence-Based Monitoring of Early-Stage Aggregation of Amyloid-β, Amylin Peptide, Tau, and α-Synuclein Proteins

**DOI:** 10.1021/acschemneuro.4c00097

**Published:** 2024-08-16

**Authors:** Yuanjie Li, Saurabh Awasthi, Louise Bryan, Rachel S. Ehrlich, Nicolo Tonali, Sandor Balog, Jerry Yang, Norbert Sewald, Michael Mayer

**Affiliations:** † Adolphe Merkle Institute, University of Fribourg, Chemin des Verdiers 4, Fribourg CH-1700, Switzerland; ‡ Department of Biotechnology, National Institute of Pharmaceutical Education and Research, Raebareli (NIPER-R), Lucknow, Uttar Pradesh 226002, India; § Department of Chemistry and Biochemistry, University of California San Diego, La Jolla, California 92093-0358, United States; ∥ CNRS, BioCIS, Bâtiment Henri Moissan, Université Paris-Saclay, 17 Av. des Sciences, Orsay 91400, France; ⊥ Bielefeld University, Department of Chemistry P.O. Box 100131, Bielefeld 33501, Germany

**Keywords:** taBODIPY, AN-SP, tau, α-synuclein, amylin, amyloid-beta (Aβ), early stage aggregates, fluorescence

## Abstract

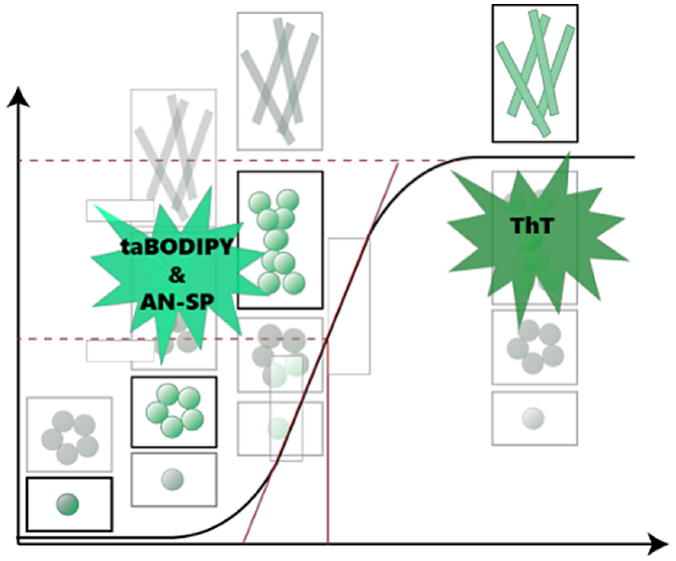

Early-stage aggregates of amyloid-forming proteins, specifically soluble oligomers, are implicated in neurodegenerative diseases such as Alzheimer’s disease, Parkinson’s disease, and Huntington’s disease. Protein aggregation is typically monitored by fluorescence using the amyloid-binding fluorophore thioflavin T (ThT). Thioflavin T interacts, however, preferentially with fibrillar amyloid structures rather than with soluble, early-stage aggregates. In contrast, the two fluorophores, aminonaphthalene 2-cyanoacrylate-spiropyran (AN-SP) and triazole-containing boron-dipyrromethene (taBODIPY), were reported to bind preferentially to early-stage aggregates of amyloidogenic proteins. The present study compares ThT with AN-SP and taBODIPY with regard to their ability to monitor early stages of aggregation of four different amyloid-forming proteins, including amyloid-β (Aβ), tau protein, amylin, and α-synuclein. The results show that the three fluorophores vary in their suitability to monitor the early aggregation of different amyloid-forming proteins. For instance, in the presence of Aβ and amylin, the fluorescence intensity of AN-SP increased at an earlier stage of aggregation than the fluorescence of ThT, albeit with only a small fluorescence increase in the case of AN-SP. In contrast, in the presence of tau and amylin, the fluorescence intensity of taBODIPY increased at an earlier stage of aggregation than the fluorescence of ThT. Finally, α-synuclein aggregation could only be monitored by ThT fluorescence; neither AN-SP nor taBODIPY showed a significant increase in fluorescence over the course of aggregation of α-synuclein. These results demonstrate the ability of AN-SP and taBODIPY to monitor the formation of early-stage aggregates from specific amyloid-forming proteins at an early stage of aggregation, although moderate increases in fluorescence intensity, relatively large uncertainties in fluorescence values, and limited solubility of both fluorophores limit their usefulness for some amyloid proteins. The capability to monitor early aggregation of some amyloid proteins, such as amylin, might accelerate the discovery of aggregation inhibitors to minimize the formation of toxic oligomeric species for potential therapeutic use.

## Introduction

Protein aggregation is associated with many human diseases, including neurodegenerative disorders such as Alzheimer’s disease (AD)^1,2^ and Parkinson’s disease^3,4^ non-neurological pathological disorders such as type 2 diabetes,^5,6^ or atrial amyloidosis.^7^ Among these diseases, AD is the most prevalent neurodegenerative disorder, characterized by irreversible, progressive memory loss and deficits in cognitive function.^8^ The presence of extracellular aggregates and plaques of amyloid-β (Aβ) peptides or intracellular neurofibrillary tangles of tau protein are the hallmarks of AD.^9^ Amyloid-β (1–42) peptides (∼4.5 kDa) found in senile plaques arise from a two-step cleavage process of the amyloid precursor protein; a process that is enzymatically catalyzed by β-secretase and γ-secretase.^10^ Tau protein (∼55 kDa), on the other hand, exists in six different isoforms based on its microtubule-binding repeats and the number of N-terminal domains.^11,12^ Since the full-length isoform of tau protein aggregates slowly, assays that examine tau aggregation commonly take advantage of aggregation-prone repeat domains of tau, such as K18 (∼15.1 kDa), which aggregate faster.^11^ Furthermore, heparin, a polyanionic inducer, accelerates tau protein aggregation *in vitro*.^11,13,14^

Growing evidence suggests that early-stage aggregates of Aβ peptide and tau protein are the most neurotoxic species in the brain of affected individuals.^15−20^ This toxic role of small aggregates is supported by the observation that the number of amyloid plaques in the brain, which are composed predominantly of mature Aβ fibrils, does not correlate well with the degree of cognitive impairment or loss of neurons and synapses.^21^ Similar observations have been made for α-synuclein (αSyn) in the context of Parkinson’s disease. Here we also monitored the aggregation of amylin (also called islet amyloid polypeptide, IAPP), which is relevent in the context of diabetes mellitus.^22,23^ Amylin is a 37-residue peptide hormone (3.9 kDa) stored in the islets of Langerhans of the pancreas.^24^ Amylin aggregation is linked to cell death of pancreatic β cells in type 2 diabetes.^25^ Moreover, IAPP oligomers have been identified as toxic species to β cells by disrupting their membranes both extra- and intracellularly.^26,27^ Finally, aggregation of αSyn protein (14.5 kDa) is associated with a group of neurodegenerative disorders known as synucleinopathies that include Parkinson’s disease,^28−30^ and toxicity of soluble oligomers of αSyn has been demonstrated *in vivo*.^29,31^

In order to study the formation of protein amyloids, several groups,^33^ including ours,^32^ have used covalent labeling or site-specific modifications of monomeric proteins.^32,33^ Single-molecule fluorescence^34^ and single-molecule stepwise photobleaching^35^ have been employed to detect oligomerization *in vitro*. However, intrinsic labeling of proteins may result in changes in aggregation kinetics or may influence the size, shape, and neurotoxicity of amyloid aggregates.^36,37^ Several groups, including ours, have demonstrated a size dependence of Aβ oligomers to induce ion channel-like ion flux in artificial membranes and plasma membranes of neuronal cells.^38^ In another study, we identified that amyloid oligomers consisting of 8- to 13-mers correlated positively with both pore formation and cytotoxicity.^39^ Moreover, using nanopores, we have characterized aggregates of both Aβ and αSyn in solution on a single-particle level.^40,41^

**Figure 1:**
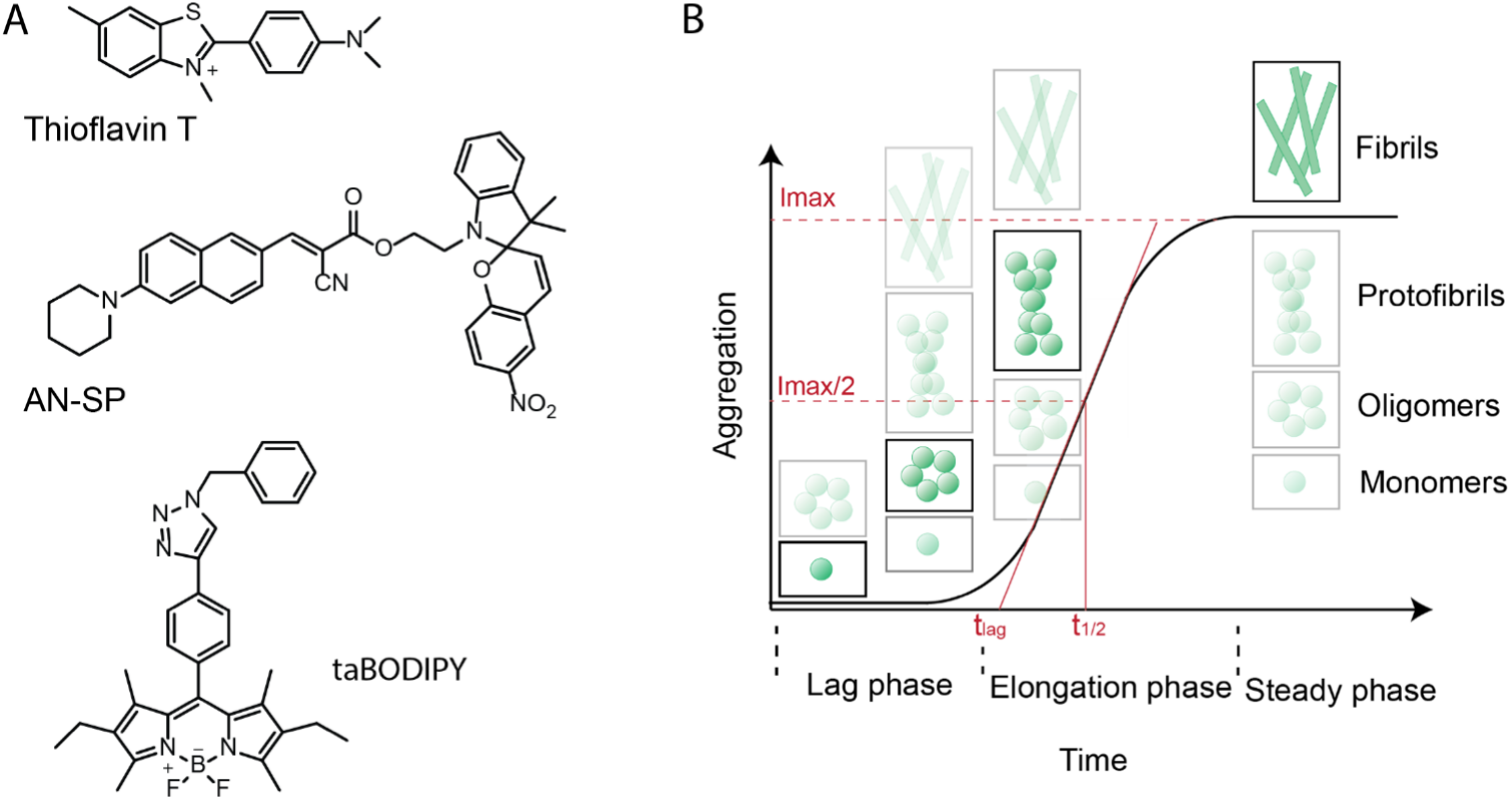
Chemical structure of amyloid-responsive fluorescent molecules and schematic illustration of protein aggregation kinetics. (A) Chemical structures of thioflavin T (ThT), amino naphthalene 2-cyanoacrylate-spiropyran (AN-SP), and triazole-containing boron-dipyrromethene (taBODIPY) fluorophores. (B) Schematic illustration of different phases (lag phase, elongation phase, and steady/saturation phase) of the protein aggregation pathway. The insets indicate the presence of various amyloid particles with changing abundances at different stages of aggregation. These particles range from monomers, oligomers, and protofibrils to fibrils; the most visible particles (i.e., the ones with the lowest opacity) are the species that are characteristic of each aggregation stage. The parameter *t*_1/2_ is represented in eq 2 and, in the simplest case, represents the time to reach half of the maximum fluorescence intensity *I*_max_. The parameter *t*_lag_ is represented in Eq 3 and signifies the duration of the lag phase.

Thioflavin T (ThT) is the most widely used fluorescent dye to detect protein aggregation to amyloids *in vitro*.^42−44,56,57^ However, ThT fails to detect early-stage aggregation, specifically the formation of oligomer species,^20−22,52^ and only exhibits an increased fluorescence emission upon binding the crossed-β-sheet structure of amyloid fibrils.^9−11,52,56^ Yang et al.^58^ and Li et al.^59^ have developed near-infrared fluorescence probes such as PTO-29 and CRANAD-102 to detect early-stage aggregates. These promising probes could selectively detect Aβ oligomers and image early-stage Aβ aggregates *in vivo*. The work presented here aims to identify and compare suitable fluorescent probes to detect early-stage aggregates of four different amyloid-forming proteins.^45,46^ To this end, we explored two fluorophores that have recently been reported to bind to early-stage aggregates of Aβ, called aminonaphthalene 2-cyanoacrylate-spiropyran (AN-SP)^47^ and triazole-containing boron-dipyrromethene (taBODIPY).^48−51^ As shown in Figure 1A, AN-SP is composed of an amino naphthalene 2-cyanoacrylate (ANCA) molecule linked to a spiropyran (SP) skeleton. Lv et al.^47^ showed that AN-SP increases its fluorescence intensity in the presence of early-stage Aβ aggregates, while Tonali et al.^48^ reported that taBODIPY also increases the fluorescence intensity in the presence of early-stage Aβ aggregates. Here, we directly compared these early stage-binding fluorophores with ThT and assessed their ability to increase their fluorescence intensity in the presence of four different amyloid proteins, Aβ(1–42), IAPP, αSyn, and K18-Tau. We show that early aggregates of Aβ may be monitored with AN-SP, as it increases its fluorescence at an earlier stage of Aβ aggregation than ThT; with the caveat, however, that the increase in fluorescence intensity is weak. In contrast, early aggregates of IAPP are best monitored by taBODIPY followed by AN-SP, and again, both fluorophores increase their fluorescence at an earlier stage of IAPP aggregation than ThT. Early aggregates of K18-tau are best monitored by taBODIPY, which increases its fluorescence at an earlier stage of tau aggregation than ThT; with the caveat, however, that the uncertainty in fluorescence intensity is larger than the one from using ThT. Finally, aggregation of αSyn can only be monitored with ThT (presumably by binding to late-stage, fibrillar aggregates); neither AN-SP nor taBODIPY show a reliable response during aggregation of αSyn. This work, therefore, reveals fluorescent probes that, compared to ThT, may detect earlier stage aggregates of Aβ (AN-SP), K18-tau (taBODIPY), and IAPP (taBODIPY), while displaying various degrees of specificity toward soluble aggregates from these different proteins.

## Results and Discussion

We compared the two fluorescent dyes AN-SP and taBODIPY with the well-established amyloid-responsive fluorophore ThT (Figure 1A) for their ability to monitor early-stage aggregation of four different amyloidogenic proteins: Aβ(1–42), K18-Tau, IAPP, and αSyn.

### Monitoring Early-Stage Aggregation of Aβ(1–42)

**Figure 2:**
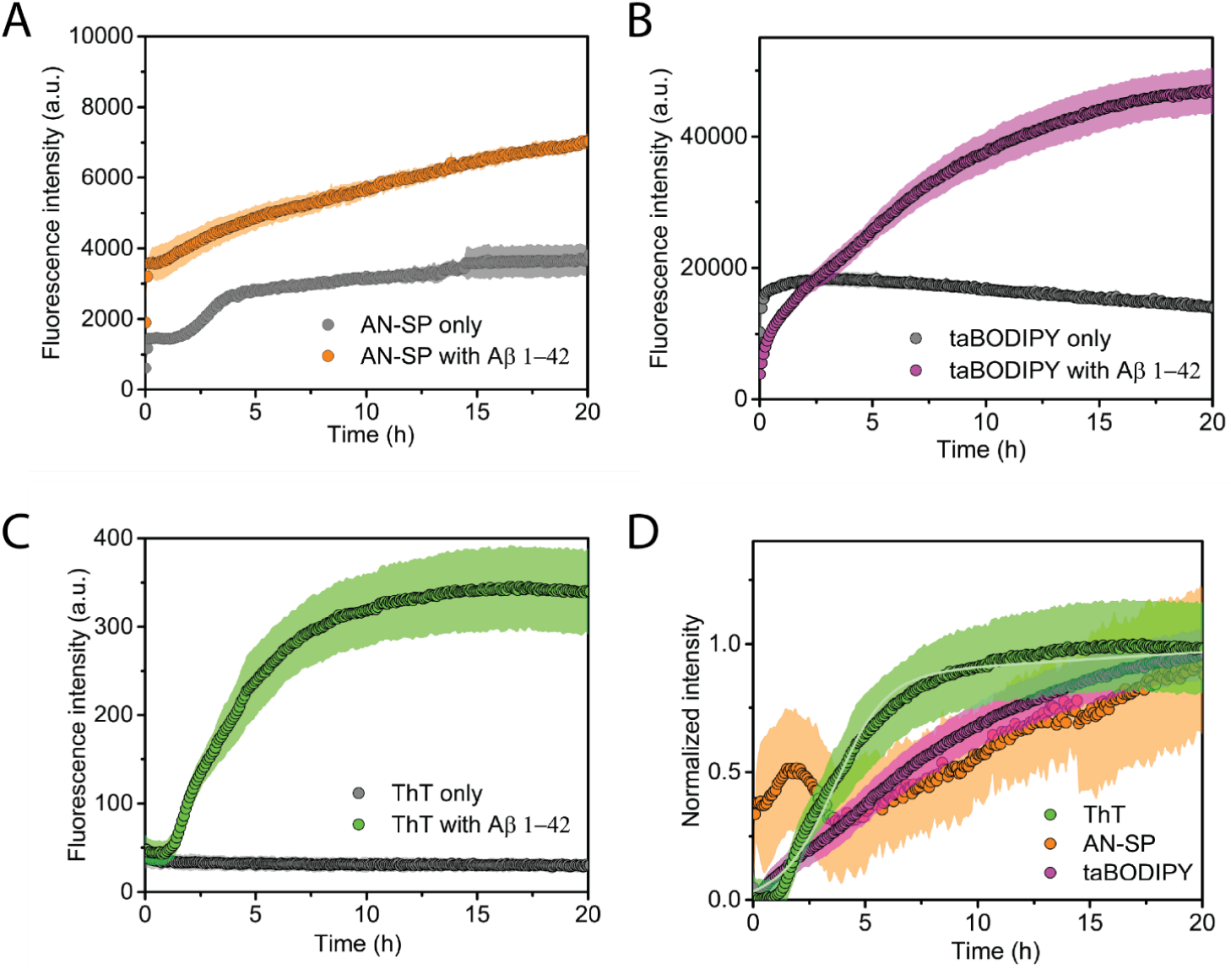
Aggregation kinetics of Aβ(1–42) peptide. (A) Aggregation of Aβ(1–42) monitored using AN-SP (orange) and blank control of AN-SP in the absence of Aβ(1–42) (gray). (B) Aggregation of Aβ(1–42) monitored using taBODIPY (purple) and blank control of taBODIPY in the absence of Aβ 1–42 (gray). (C) Aggregation of Aβ(1–42) monitored using ThT (green) and blank control of ThT in the absence of Aβ(1–42) (gray). (D) Normalized aggregation curves after subtracting the blank control for AN-SP, taBODIPY, and ThT. The light green curve shows the results of fitting eq 2 to the fluorescence intensity values over time. The AN-SP and taBODIPY data of Aβ aggregation are not suitable for fitting with the simple model underlying Eq 2 due to the complex aggregation kinetics of the AN-SP data and the high fluorescence intensity of the taBODOIPY data of the dye only at the early stage of aggregation. In the case of the taBODIPY data, the higher fluorescence intensities from the fluorophore-only control compared to those of the sample with Aβ at the beginning of the aggregation experiment severely limit the usefulness of taBODIPY for early-stage monitoring of aggregation. Each data point shown is the average of at least three measurements. In all four panels, the shaded region shows the standard deviation for each data set from at least three measurements. The regions shaded in gray for the blank controls are small and sometimes not visible.

Figure 2 shows the aggregation kinetics of the Aβ peptide in the presence of AN-SP (Figure 2A), taBODIPY (Figure 2B), or ThT (Figure 2C), along with the corresponding blank controls of the fluorescence of each fluorophore in the absence of amyloid protein. In the presence of ThT, we observed the characteristic sigmoidal aggregation kinetics (Figure 1B) of Aβ as expected. Sigmoidal aggregation kinetics^52^ includes the lag phase, elongation phase, and plateau phase, suggesting that the aggregation starts from the predominantly monomeric state in the absence of protofibrils.^58,59^ Both AN-SP and taBODIPY increased their fluorescence intensity at an early stage of Aβ aggregation, while ThT fluorescence still indicated the lag phase. This result confirms earlier reports by Lv et al.^47^ and Tonali et al.^48^ that these two fluorophores are able to detect early-stage aggregates of Aβ. To compare the ability of all three fluorophores to monitor the aggregation of Aβ at various stages of aggregation, we subtracted the fluorescence increase of the fluorophores only and normalized the fluorescence intensity values obtained for all three dyes (Figure 2D). The results show that the fluorescence intensity of AN-SP increases earlier than the intensity of taBODIPY and that the intensity from both of these fluorophores increases earlier than the one of ThT. We noticed that the lag phase observed with AN-SP is significantly shorter than that observed with ThT (Figure 2D). In fact, monitoring aggregation of Aβ(1–42) with AN-SP leads to an immediate increase in fluorescence intensity, which is in agreement with the results from Lv et al.^47^

Figure 2 shows that the control experiments with AN-SP and taBODIPY only, that is, in the absence of amyloids, also led to an increase in fluorescence intensity over time. This increase might indicate self-aggregation of the dyes. In fact, dynamic light-scattering experiments revealed the presence of aggregates of AN-SP in Aβ aggregation buffer with AN-SP only (i.e., in the absence of Aβ) (see Figure S1). The presence of such aggregates raises the concern that they may accelerate the nucleation of amyloid oligomers. Experiments with various concentrations of AN-SP or taBODIPY and two different concentrations of DMSO in the solution supported the hypothesis of limited solubility of AN-SP and taBODIPY (see Figure S2). To determine possible effects of the presence of aggregated AN-SP or taBODIPY fluorophores on the aggregation kinetics of Aβ(1–42), we carried out aggregation assays of Aβ peptide using ThT in the absence or presence of a high concentration (12.5 μM) of AN-SP or taBODIPY (for the aggregation assays in this work, we actually used 4 μM of AN-SP or 1 μM of taBODIPY) (see Figures S3 and S4). Unpaired two-sample *t-*tests revealed no difference in the aggregation kinetics. In other words, the aggregation of Aβ(1–42) monitored by ThT fluorescence in the presence of AN-SP or taBODIPY was not significantly different from monitoring Aβ aggregation with ThT only. Therefore, we found no evidence that the presence of AN-SP or taBODIPY accelerated the aggregation of Aβ(1–42), rather their fluorescence increased at an earlier stage of Aβ(1–42) aggregation than the fluorescence of ThT, as reported previously.^47 −51^

It is important to note the limitations of AN-SP and taBODIPY in detecting the early stages of Aβ aggregation. Figure 2B shows the surprising result that the fluorescence intensity of taBODIPY in the presence of Aβ is lower than the intensity of BODIPY only in the early stage of aggregation. Considering that the triplicate results are consistent with each other, taBODIPY is, hence, not a reliable indicator during the initial 2 h of Aβ aggregation. In addition, since the fluorescence intensity reaches only about twice that of the control experiment with AN-SP only, the usefulness of AN-SP for detecting Aβ aggregation may also be limited for certain applications.

The aggregation of amyloid proteins can vary from batch to batch, which may affect the detection capabilities of AN-SP or taBODIPY for early-stage aggregation. To explore this effect, we performed an aggregation experiment using a fresh batch of Aβ(1–42) peptide that was prepared using a different method (SI Note 2). Although this preparation contained likely Aβ aggregates before the start of the aggregation process, the fluorescence intensity of AN-SP still increased faster during the first 3 h of aggregation than the fluorescence intensity of ThT (see Figure S6). The results are therefore consistent with those in Figure 2.

### Monitoring Early-Stage Aggregation of K18-Tau

**Figure 3:**
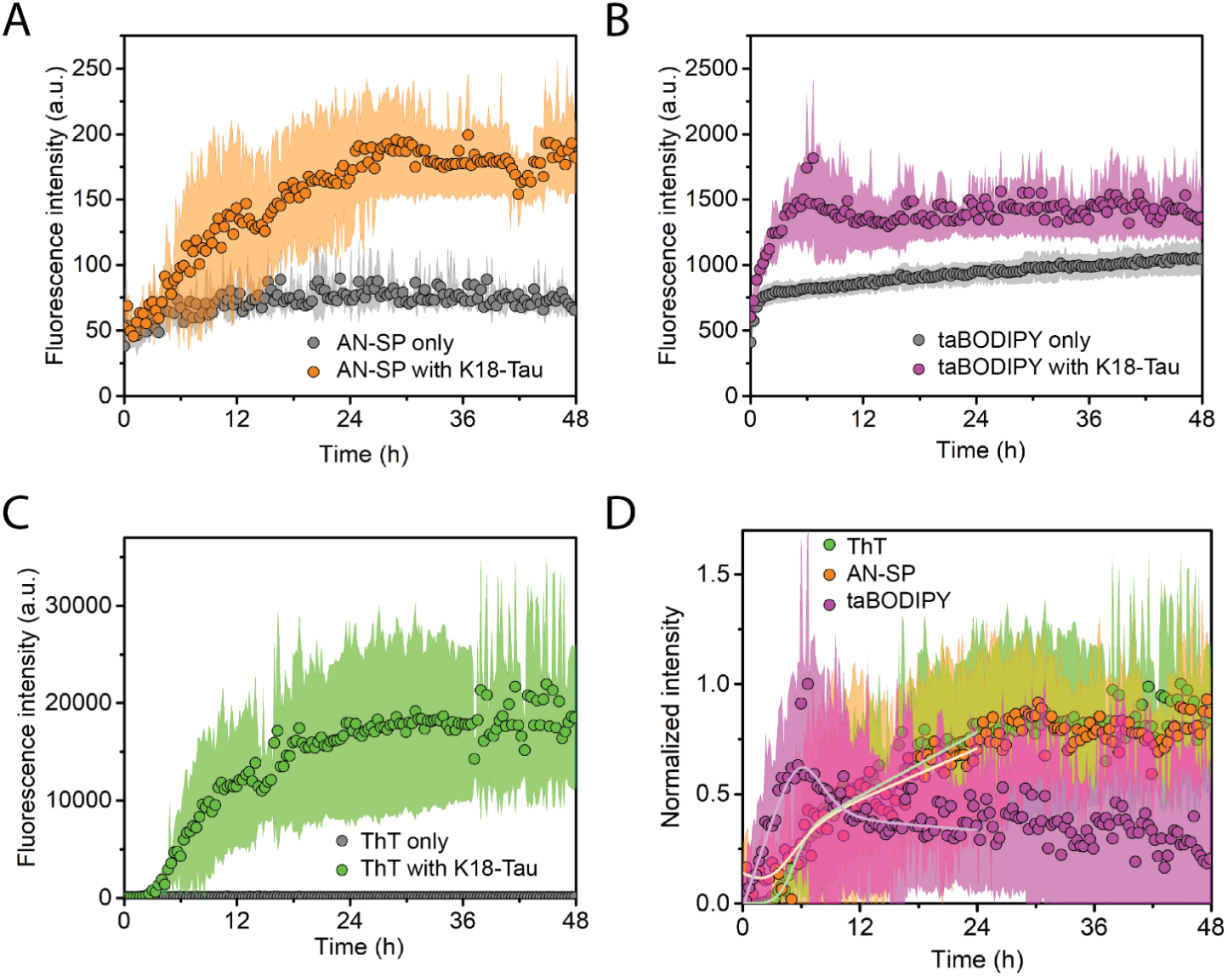
Aggregation kinetics of K18-tau protein. (A) Kinetics of K18-tau aggregation monitored using AN-SP (orange) along with the blank control of AN-SP without K18-Tau (gray). (B) Kinetics of K18-tau aggregation monitored by taBODIPY (purple) along with the blank control without protein (gray). (C) Kinetics of K18-tau aggregation monitored by ThT (green) and ThT fluorescence of the blank control (gray). (D) Comparison of aggregation kinetics of AN-SP, taBODIPY, and ThT after subtracting the blank control and normalization. The light orange curve, light purple curve, and light green curve show the results of fitting eq 2 to the data. The data points shown as filled circles are the average of at least three different measurements. The shaded region shows the standard deviation of each data set. The shaded gray region for the ThT blank control group is not always visible.

We used a fast-aggregating isoform of tau, that is, K18-Tau, containing four microtubule-binding repeats, in this study. Figure 3 shows the aggregation kinetics of K18-Tau in the presence of AN-SP (Figure 3A), taBODIPY (Figure 3B), or ThT (Figure 3C) along with the corresponding blank controls of the fluorescence of each fluorophore in the absence of K18-Tau. In the presence of ThT, the kinetics of K18-tau aggregation follows characteristic sigmoidal aggregation kinetics, which includes the lag phase, elongation phase, and plateau phase. These results suggest that aggregation starts from the predominantly monomeric state in the absence of photofibrils.^52^ In the presence of AN-SP, we observed similar aggregation kinetics compared to the aggregation kinetics monitored by ThT. The increase in fluorescence of AN-SP only in Tau aggregation buffer is less pronounced than in the aggregation buffers for the other amyloids; one plausible explanation may be that slight differences in the solubility of AN-SP in these different buffers contribute to these differences. However, the taBODIPY fluorescence intensity started to increase earlier during K18-Tau aggregation compared to ThT fluorescence, revealing for the first time that taBODIPY is able to detect early-stage aggregates of K18-Tau.^63^ To compare the ability of these fluorophores to monitor the aggregation of K18-Tau at various stages of aggregation, we again normalized the fluorescence intensity values after subtracting the corresponding blank control fluorescence intensity values in Figure 3D. The results show that the fluorescence intensity of taBODIPY increases at an earlier stage of Tau aggregation than the intensity of AN-SP and ThT and that the intensity of AN-SP and ThT shows a similar dependence with time. The concentration of oligomers typically shows a maximum at the early stages of protein aggregation.^64^ While the data in Figure 3B do not clearly show such a maximum, the data after subtracting the fluorescence intensity from taBODIPY-only and after normalization show a maximum followed by a decrease in fluorescence intensity. The uncertainty of these data is considerable due to error propagation during the subtraction of the taBODIPY-only signal and normalization, however, the curve fit to the intensity averages clearly reflects the expected behavior.

Table 1 compares the results from kinetic analyses of aggregation of K18-Tau by fitting eq 2 to the normalized fluorescence intensity of each of the three fluorophores in separate microvials for aggregation. These fitting procedures revealed values of the lag time (*t*_lag_) and half-time (*t*_1/2_) during the aggregation of K18-Tau using AN-SP, taBODIPY, or ThT for monitoring aggregation. Based on unpaired two-sample *t*-tests to compare the difference of K18-tau aggregation kinetics monitored using AN-SP or taBODIPY as a fluorophore to the kinetics monitored using ThT as a fluorophore, the results in Table 1 show the shortest lag phase of K18-tau aggregation for taBODIPY, which is significantly different from both ThT and AN-SP. However, the difference in the lag time between AN-SP and ThT was not significantly different.

**Table 1:** Comparison of the Half-Time *t*_1/2_ and Lag Time *t*_lag_ of Time**-**Dependent Aggregation of Four Different Proteins Using Three Different Fluorophores ThT, taBODIPY, and AN-SP^a^

	**ThT**		**taBODIPY**		**AN-SP**	
proteins	*t*_1/2_ (min)	*t*_lag_ (min)	*t*_1/2_ (min)	*t*_lag_ (min)	*t*_1/2_ (min)	*t*_lag_ (min)
Aβ(1–42)	222.1 ± 25.6	83.7 ± 19.7	-	-	-	-
K18-Tau	301.4 ± 5.7	199.4 ± 7.9	256.6 ± 48.8	60.9 ± 20.8	63.9 ± 104.1	240.1 ± 68.4
			*p* = 0.1871	*p* = 0.0004	*p* = 0.0169	*p* = 0.3638
amylin	136.5 ± 0.8	71.9 ± 1.5	46.6 ± 1.4	0.0 ± 0.0	64.0 ± 1.4	27.9 ± 2.1
			*p* = 0.0001	*p* = ∼0	*p* = 0.0001	*p* = 0.0001
αSyn	1547.4 ± 13.9	1094.4 ± 19.3	-	-	-	-

a The *p* values from unpaired two-sample *t*-tests in the table convey the significance level of the *t*_1/2_ and *t*_lag_ values obtained with ThT for monitoring aggregation compared to the *t*_1/2_ and *t*_lag_ values obtained with either taBODIPY or AN-SP for monitoring aggregation. We consider a comparison to be significantly different for *p* values ≤0.05.

Meaningful estimation of *t*_lag_ and *t*_1/2_ values from the fluorescence kinetics of amyloid aggregation, as shown in Table 1, requires that the fluorescence intensity be proportional to the concentration of amyloid aggregates. Therefore, we compared the relative fluorescence intensity of AN-SP or taBODIPY in the presence of different concentrations of early-stage K18-Tau, Aβ(1–42), and IAPP aggregates. The results show that the fluorescence intensity of AN-SP and taBODIPY is proportional to the concentrations of preformed aggregates (see Figure S5).

Similar to the case of Aβ(1–42), we tested a possible effect of the taBODIPY dye on the aggregation kinetics of tau protein. As with Aβ(1–42), we carried out *in vitro* aggregation assays using ThT in the presence of a high concentration (12.5 μM) of taBODIPY. Unpaired two-sample *t-*tests revealed no significant difference in the kinetics of K18-Tau as measured by ThT fluorescence when either ThT only was present or ThT was present together with either AN-SP or taBODIPY.

### Monitoring Early-Stage Aggregation of IAPP

**Figure 4:**
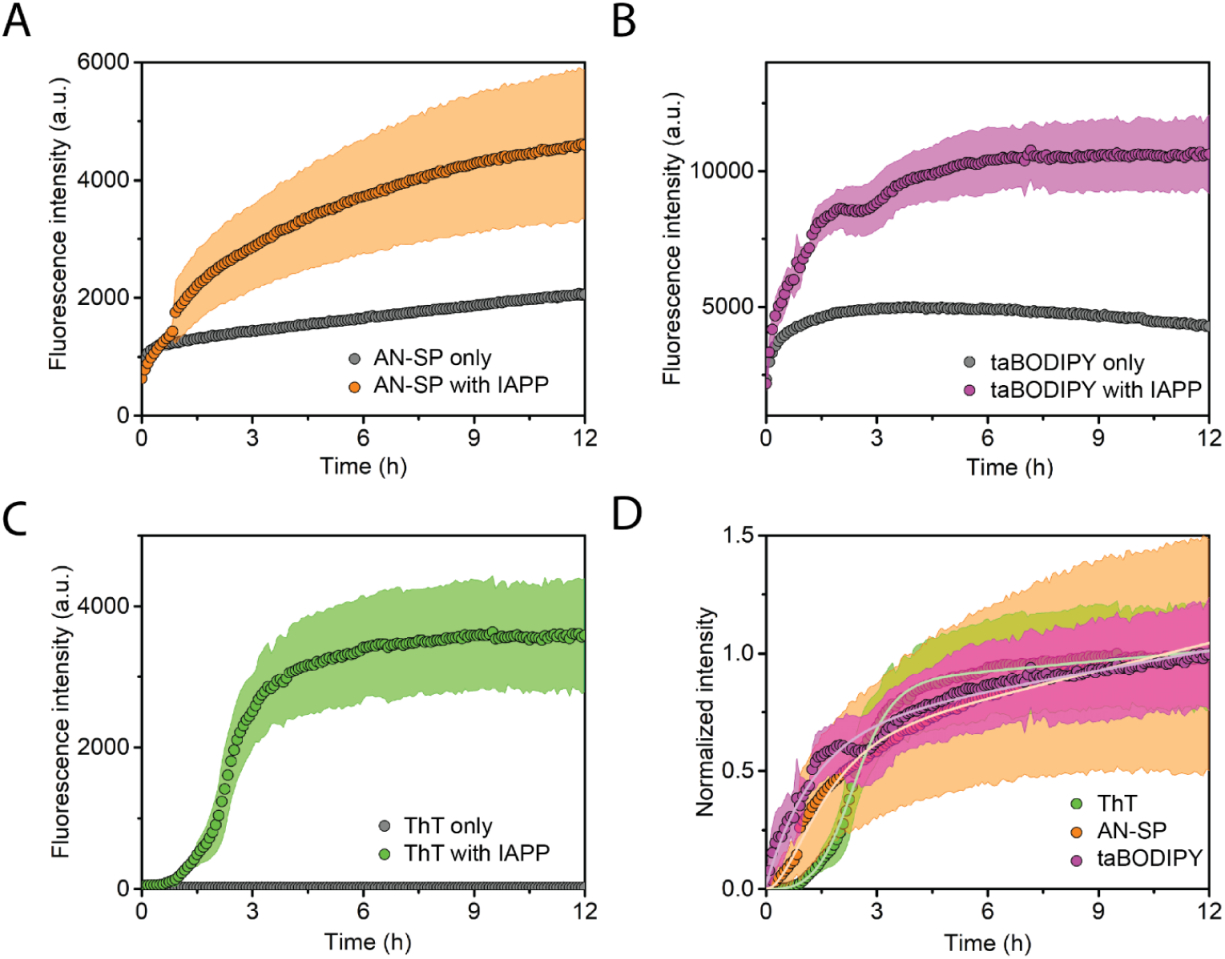
Aggregation kinetics of islet amyloid polypeptide (IAPP). (A) Kinetics of IAPP aggregation monitored using AN-SP (orange) along with the blank control of AN-SP without IAPP (gray). (B) Kinetics of IAPP aggregation monitored using taBODIPY (purple) along with the blank control (gray). (C) Kinetics of IAPP aggregation monitored using ThT (green) along with the blank control (gray). (D) Comparison of normalized aggregation curves for AN-SP, taBODIPY, and ThT after subtracting the fluorescence intensity of the blank control. The light orange curve, light pink curve, and light green curve result from fits of eq 2 to the fluorescence data. Data points shown are the averages of at least three different repeats. The shaded region shows the standard deviation of each data set. The shaded region for the blank control group is not visible.

Figure 4 shows the aggregation kinetics of amylin peptide, also called IAPP, in the presence of AN-SP (Figure 4A), taBODIPY (Figure 4B), or ThT (Figure 4C) along with the corresponding black controls of the fluorescence of each fluorophore in the absence of amyloid proteins. Before the aggregation kinetics experiments, we treated IAPP with HFIP to solubilize and monomerize possible oligomeric or fibrillar IAPP in peptide preparation, followed by removal of HFIP prior to aggregation studies. Figure 4 shows that with ThT, we observed the characteristic sigmoidal aggregation curve of IAPP as expected^60,61^ indicating that aggregation started from the predominantly monomeric state of IAPP. Both AN-SP and taBODIPY increased their fluorescence intensity at an early stage of IAPP aggregation, while the ThT fluorescence still indicated the lag phase. These results demonstrate—for the first time—that both AN-SP and taBODIPY are able to detect early aggregates of IAPP. Again, Figure 4D compares the ability of the three fluorophores to monitor the aggregation of IAPP by normalizing the fluorescence intensity values after subtracting the blank fluorescence of the fluorophore only. The results show that the fluorescence intensity of taBODIPY increases earlier than the intensity of AN-SP and that the intensity from both of these fluorophores increases earlier than the fluorescence intensity of ThT.

Note, the lack of a plateau of fluorescence intensity of AN-SP in the steady phase of aggregation of Aβ and IAPP during the time frame of the experiment may originate from the formation of amyloid oligomers by secondary nucleation mechanisms, which results in a rapid increase in fluorescence during the elongation phase and a slow, steady increase during the plateau phase.^65^

In Table 1, we present a statistical comparison of the results from analyzing aggregation kinetics of IAPP by fitting eq 2 to the normalized fluorescence intensity data of AN-SP, BODIPY, and ThT. Aggregation of IAPP shows no lag phase when taBODIPY is used as a fluorophore and a lag phase that is significantly shorter than that of ThT when AN-SP is used as a fluorophore instead of ThT. Although the lack of a clear plateau phase in Figures 2A and 4A complicates the comparison between the normalized profiles recorded with different dyes, the choice of fitting with eq 2, as well as a focus on early stages of aggregation, may still provide a useful comparison of these data with all other data in this early stage.

### Monitoring Early-Stage Aggregation of α-Synuclein

**Figure 5:**
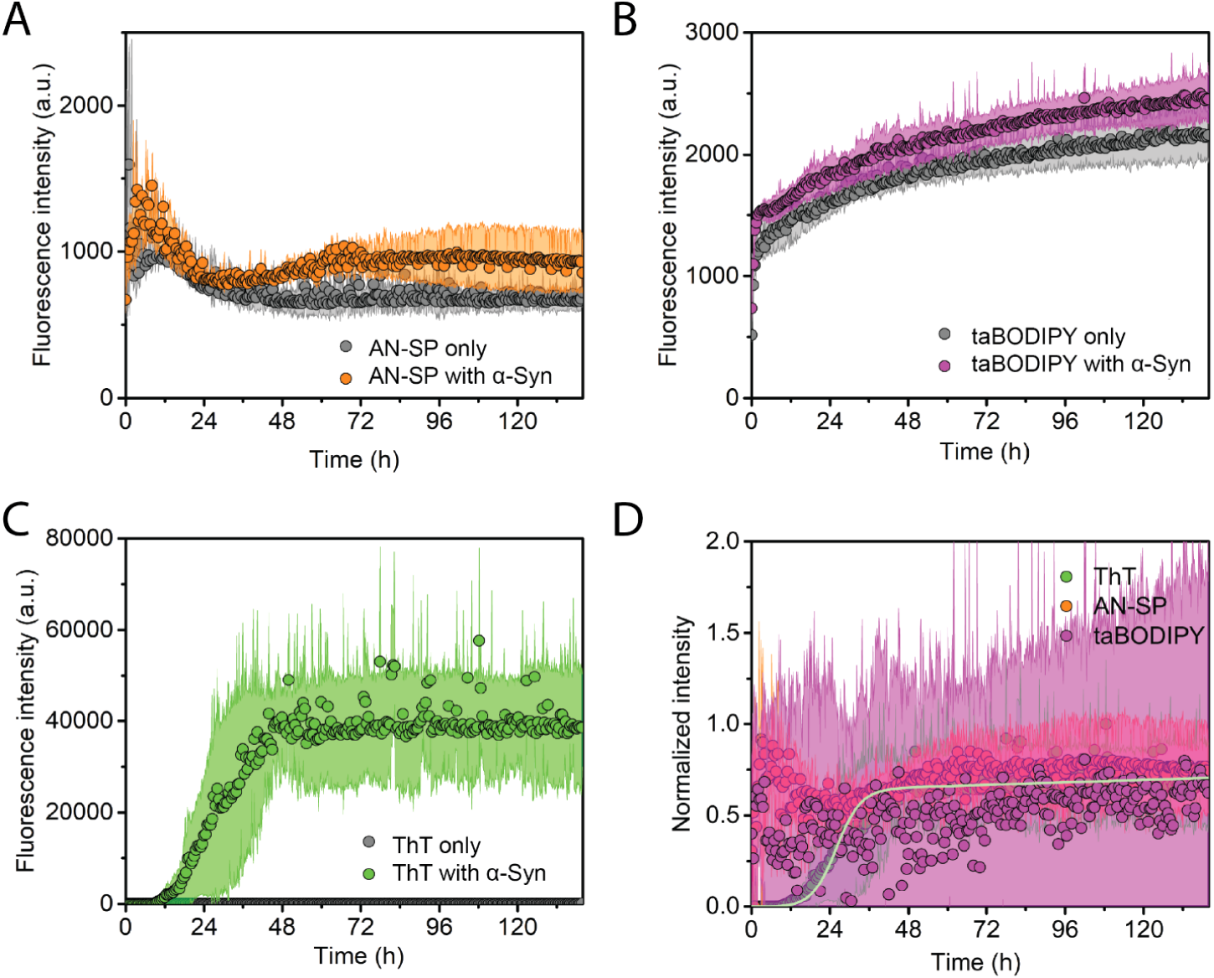
Aggregation kinetics of α-synuclein protein. (A) Kinetics of αSyn aggregation monitored in the presence of AN-SP (orange) along with blank control samples of AN-SP without αSyn (gray). (B) Kinetics of αSyn aggregation monitored using taBODIPY (purple) along with blank control samples of AN-SP without αSyn (gray). (C) Kinetics of αSyn aggregation monitored using ThT (green) along with blank control samples of AN-SP without αSyn (gray). (D) Normalized fluorescence intensity after subtracting the blank control for AN-SP, taBODIPY, and ThT. The light green curve shows the amyloid aggregation fitting results for the ThT assays. The data points shown as filled circles are the average of at least three measurements. The shaded region shows the standard deviation of each data set. The shaded gray region for the ThT blank control is not visible.

Finally, we examined the ability of AN-SP and taBODIPY to detect early-stage aggregates of αSyn. Figure 5 shows the aggregation kinetics of αSyn protein in the presence of AN-SP (Figure 5A), taBODIPY (Figure 5B), or ThT (Figure 5C) fluorophores. In contrast to the expected sigmoidal increase in fluorescence intensity of ThT as a result of αSyn aggregation, we observed no clear increase in fluorescence intensity when we attempted to monitor αSyn aggregation with the AN-SP or taBODIPY fluorophore. Hence, both AN-SP and taBODIPY are not suitable to detect any stage of αSyn aggregation. This result shows that the binding of AN-SP or taBODIPY to aggregates of the four amyloid-forming peptides and proteins studied here is selective for Aβ(1–42), K18-Tau, and IAPP, while both fluorophores do not bind aggregates of αSyn. In contrast, ThT binds to aggregates of all four amyloids but at a stage of aggregation later than that of AN-SP or taBODIPY.

In summary, we observed that both AN-SP and taBODIPY were able to interact with early-stage aggregates of the Aβ peptide, K18-tau protein, and IAPP. By using four different amyloid-forming proteins, we were able to observe preferential binding of AN-SP and taBODIPY to early-stage aggregation of amyloid proteins. For instance, AN-SP performed best with Aβ, showing a modest increase in fluorescence intensity at an early stage, whereas taBODIPY performed best with K18-tau protein early-stage aggregates. Protein specificity of AN-SP and taBODIPY dyes may enable the use of these dyes for selective detection of soluble amyloid aggregates in biological samples, for example, by comparing the response of these dyes with the response of ThT, as done in this work.

## Conclusions

Current research focuses on early detection of amyloid aggregates in neurodegenerative diseases. Here, we explored the ability of two fluorescent dyes to monitor the early-stage aggregation of four amyloidogenic proteins. Key observations of this comparative analysis of monitoring early stages of amyloid aggregation are as follows: (1) AN-SP dye is capable of detecting Aβ(1–42) early-stage aggregates with a modest increase in fluorescence. (2) taBODIPY responds early to K18-tau protein aggregation. (3) Both AN-SP and taBODIPY exhibit increases in fluorescence intensity at an earlier stage than ThT during IAPP aggregation, with taBODIPY being the first. (4) As opposed to ThT, neither AN-SP nor taBODIPY increase their fluorescence during aggregation of αSyn. This study demonstrates the potential usefulness of AN-SP and taBODIPY to detect early-stage protein aggregation of Aβ(1–42), K18-Tau, and IAPP. The results show specificity in the sense that an increase in fluorescence of ThT combined with no increase in fluorescence of AN-SP or taBODIPY may indicate aggregation of αSyn. We hope that these results will inform researchers about the choice of the most appropriate fluorescent dyes depending on the amyloid-forming proteins under study.

## Methods

### Materials

IAPP was obtained as a lyophilized powder from AnaSpec (Cat. No. AS-60804). K18-tau protein was obtained from Novus Biologicals (Cat. No. SP-496-100). Aβ(1–42) peptide was purchased from Bachem (Cat. No. 4014447.1000) as a lyophilized powder. Recombinant α-synuclein was obtained from rPeptide (Cat. No. S-1001-2). 1,1,1,3,3,3-Hexafluoro-2-propanol (HFIP) was purchased from Sigma-Aldrich (Cat. No. 18127). Thioflavin T (ThT) (Cat. No. T3516) was purchased from Sigma, USA, dissolved, and aliquoted in water. AN-SP was synthesized as previously reported.^47^ taBODIPY was synthesized in the Sewald Lab as previously reported.^48^ Beads with 0.7–1.2 mm diameter (Cat. No. G1152) were obtained from Sigma, USA. Nonbinding 384-well plates (Cat. No. 3544) were obtained from Corning.

### *In Vitro* Aggregation Assay

We carried out *in vitro* aggregation assays of Aβ peptide, K18-Tau, αSyn, and IAPP using a Cytation 5 plate reader (Biotek). All the fluorescence measurements were carried out in black 384-well plates with clear flat bottom under continuous shaking. The shaking, however, is paused during the fluorescence measurement. Excitation/emission wavelengths were set to 440 nm/490 nm for ThT, 430 nm/535 nm for AN-SP, and 500 nm/545 nm for taBODIPY. Fluorescent dyes were used with a final concentration of 10 μM of ThT, 4 μM of AN-SP, and 1 μM of taBODIPY. All experiments resulting in data for each individual figure were performed with aliquots of protein solution from the same batch of protein. Before comparing aggregation kinetics monitored with different fluorophores, we normalized the fluorescence data in the following way: First, we subtracted the fluorescence intensity of the blank control from the fluorescence intensity with the peptide at every time point, yielding the blank-corrected fluorescence *F*_c_(*t*). We then calculated the normalized fluorescence intensity *F*_n_(*t*) in the following way:
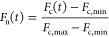
1

We determined the kinetics of aggregation based on the following parameters in eq :2(53,56,57)
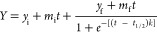
2

Here, *Y* is the fluorescence intensity as a function of time *t*, *y*_i_ and *y*_f_ are the intercepts of the initial and final fluorescence values with the *y*-axis, *m*_i_ and *m*_f_ are slopes of the fluorescence intensity during the initial aggregation phase (i.e., the lag phase) and the final aggregation phase (i.e., the steady phase), *t*_1/2_ is the time needed to reach halfway through the elongation phase, and *k* is the elongation rate constant. The lag time is usually defined as given by eq :3(53,56,57)
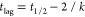
3

### *In Vitro* Aggregation of Aβ(1–42)

We prepared monomeric Aβ(1–42) as explained earlier.^54^ Briefly, 1 mg of lyophilized Aβ(1–42) was dissolved in 1 mL of 6 M guanidine hydrochloride and incubated for 5 min. Aβ solutions were centrifuged at 16000 g for 15 min at 4 °C and injected onto a Superdex 75 Increase 10/300 GL column (GE Healthcare), which had been pre-equilibrated with 10 mM Tris, pH 7.4, on an AKTA pure chromatography system (GE Healthcare). Fractions of 12–14 mL corresponding to monomeric Aβ(1–42) peptide were pooled, aliquoted, flash-frozen in liquid nitrogen, and stored at −80 °C. We determined the concentration of purified Aβ(1–42) monomer based on an absorbance measurement at 280 nm with an extinction coefficient of 1.49 mM^–1^cm^–1^.^55^*In vitro* aggregation experiments of Aβ (10 μM) were carried out using aggregation buffer consisting of 10 mM Tris with a pH of 7.5, 100 mM NaCl, and 5% (v/v) DMSO. The fluorescence intensity measurements were carried out in 384-well plates at 37 °C, and 300 rpm shaking speed during incubation using a BioTek Cytation 5 Plate reader. All of the experiments were performed with the same batch of aliquots.

### *In Vitro* Aggregation of K18-Tau

We carried out *in vitro* aggregation assays of K18-Tau (20 μM) using aggregation buffer consisting of 1 × phosphate buffered saline (PBS) with a pH of 7.4 with 5% (v/v) DMSO and 1 mM Tris(2-carboxyethyl)phosphine (TCEP). We used heparin as an inducer at a concentration of 0.5 mg/mL. We used a single-glass bead ranging in diameter from 0.7 to 1.2 mm per well as reported previously.^56^ Fluorescence intensity measurements were carried out at 37 °C, 500 rpm shaking speed. All the experiments were performed with the same batch of aliquots.

### *In Vitro* Aggregation of IAPP

We used HFIP treatment to monomerize IAPP before *in vitro* aggregation assays as explained earlier.^57^ Briefly, lyophilized IAPP was dissolved in HFIP at a concentration of 1 mg/mL and vortexed for 2 min.^62^ Small aliquots (10 μL) were transferred to 0.5 mL microvials and placed in the hood to dry overnight. To ensure maximum removal of HFIP from IAPP aliquots, the microvials were placed in a desiccator under vacuum for 1 h, followed by blowing a gentle stream of nitrogen (N_2_) gas over the open vials. The clear film of peptide formed on the surface of the tube was then stored at −80 °C until use. The IAPP films were dissolved in 125 μL DMSO to a concentration of 200 μM. *In vitro* aggregation assays were carried out using IAPP at a final concentration of 10 μM in 1 × PBS buffer. Measurements were taken every 5 min with 3000 min in total at 25 °C and 300 rpm shaking speed between readings. All the experiments were performed with the same batch of aliquots.

### *In Vitro* Aggregation of α-Synuclein

We prepared 100 μL of a solution containing 100 mg/mL αSyn from Enzo Life Science and stored 5 μL aliquots at −80 °C. We diluted these 5 μL aliquots to a final αSyn concentration of 10 μM in 20 mM Tris-HCl buffer, pH 7.5, containing 100 mM NaCl and 1 mM MgCl_2_ with 10 μM ThT, 4 μM AN-SP, or 1 μM taBODIPY, respectively. Single-glass beads with a diameter of 0.7–1.2 mm were added to each well in the microwell plates, and experiments were carried out with 300 rpm shaking between fluorescence readings at 37 °C and taken every 15 min for 9000 min (∼6 days). All the experiments were performed with the same batch of aliquots.

### Amyloid Fibril Preparation

Two 100 μL of a solution of Aβ(1–42) (20 μM) were prepared in Aβ aggregation buffer. The protein samples were incubated at 37 °C for 20 h. The same fibril sample preparation procedure was employed for K18-Tau (40 μM) and IAPP (20 μM). Subsequently, AN-SP or taBODIPY were added to the samples at final concentrations of 4 and 1 μM, respectively. Different concentrations of protein were achieved by diluting the sample with the aggregation buffer containing the same concentration of AN-SP or taBODIPY. Fluorescence emission spectra were obtained using a Fluorolog-3 (HORIBA Scientific) spectrofluorometer with excitation wavelengths of 430 and 500 nm, respectively. The relative fluorescence intensity was calculated by dividing the peak fluorescence intensity in the presence of proteins by the value in the absence of proteins.

### DLS Measurements for AN-SP Aggregates

DLS data were collected at a constant temperature (23 °C) on a commercial goniometer instrument (3D LS Spectrometer, LS Instruments AG, Switzerland). The primary beam was formed by a linearly polarized and collimated laser beam (Cobolt 05-01 diode pumped solid state laser, λ = 660 nm, *P*max = 500 mW), and the scattered light was collected by single-mode optical fibers equipped with integrated collimation optics. The incoming laser beam passed through a Glan–Thompson polarizer with an extinction ratio of 10–6, and another Glan–Thompson polarizer, with an extinction ratio of 10–8, was mounted in front of the collection optics. DLS data were collected at the scattering angle of 90°. To construct the intensity autocorrelation function *g*_2_(*t*), the collected light was coupled into two APD detectors via laser-line filters (PerkinElmer, Single Photon Counting Module), and their outputs were fed into a two-channel multiple-tau correlator. To improve the signal-to-noise ratio and to eliminate the impact of detector afterpulsing on *g*_2_(*t*) at early lag times, these two channels were cross-correlated. The autocorrelation functions were regressed by reparametrized gamma distributions (also known as Schulz–Zimm distribution).

## Supporting Information Available

Detailed information about the control experiment to determine the effect of the taBODIPY or AN-SP fluorophores on the aggregation kinetics of amyloid proteins (Note 1), detailed information about the control experiment to explore the variations in different batches of Aβ peptide preparation (Note 2), particle size distribution of AN-SP in Aβ aggregation (Figure S1), fluorescence intensity of various concentrations of AN-SP or taBODIPY in the presence of 0% or 5% DMSO in the solution (Figure S2), the effect of AN-SP or taBODIPY dye on the aggregation kinetics of Aβ peptide (Figure S3), the effect of AN-SP or taBODIPY dye on the aggregation kinetics of K18-tau protein (Figure S4), relative fluorescence intensity of AN-SP or taBODIPY as a function of the concentration of aggregates of Aβ, IAPP, or tau (Figure S5), aggregation kinetics of Aβ(1–42) peptide from a batch different from the one used in the main text (Figure S6), half-time *t*_1/2_ and lag time *t*_lag_ of Aβ(1–42) aggregation using ThT assays in the presence or absence of taBODIPY or AN-SP (Table S1), and half-time *t*_1/2_ and lag time *t*_lag_ of Aβ(1–42) aggregation using ThT assays in the presence or absence of taBODIPY or AN-SP (Table S2) (PDF)
