# Supplementary material for: Fluorescence-Based Monitoring of Early-Stage Aggregation of Amyloid-β, Amylin Peptide, Tau, and α-Synuclein Proteins
Source: ACS Chem Neurosci. 2024 Aug 16;15(17):3113–23. doi: 10.1021/acschemneuro.4c00097 (PMC11378287; doi:10.1021/acschemneuro.4c00097)
Supplement: Supplementary file 1 — cn4c00097_si_001.pdf [file cn4c00097_si_001.pdf]

Supporting Information

Fluorescence-based Monitoring of Early-Stage Aggregation of Amyloid-β, Amylin peptide, Tau and α-Synuclein Proteins

Yuanjie Li[1], Saurabh Awasthi[1, 2*], Louise Bryan[1], Rachel S. Ehrlich[3], Nicolo Tonali[4], Sandor Balog[1], Jerry Yang[3], Norbert Sewald[5], Michael Mayer[1*]

[1] Adolphe Merkle Institute, University of Fribourg, Chemin des Verdiers 4, CH-1700 Fribourg, Switzerland

[2] Department of Biotechnology, National Institute of Pharmaceutical Education and Research, Raebareli (NIPER-R), Lucknow-226002, Uttar Pradesh, India

[3] University of California San Diego, Department of Chemistry and Biochemistry, La Jolla, CA, 92093-0358 USA

[4] CNRS, BioCIS, Bâtiment Henri Moissan, Université Paris-Saclay, 17 Av. des Sciences, Orsay, 91400 France

[5] Bielefeld University, Department of Chemistry, P. O. Box 100131, 33501 Germany

**Corresponding Author Email ID: rf.saurabh.awasthi@niperrbl.ac.in, michael.mayer@unifr.ch*

Supplementary Note 1:

To investigate a possible effect of the taBODIPY or AN-SP fluorophores on the aggregation kinetics of amyloid proteins, we carried out *in vitro* assays of aggregation of Aβ peptide and K18-Tau protein using ThT dye in the presence of the following concentrations of taBODIPY or AN-SP: 1 µM, 5 µM, and 12.5 µM. **Figure S3 and, in particular Table S1,** show the results of the aggregation assays using Aβ peptide. The results from fitting equation 2 to the data show no significant effect of taBODIPY or AN-SP on the aggregation kinetics of the Aβ peptide.

Similarly, **Figure S4 and, in particular Table S2,** show the results of the aggregation assays using K18-Tau protein. Even in the presence of high taBODIPY or AN-SP concentrations of 12.5 µM, and hence more than 3-times their concentration used in this study, the aggregation kinetics of K18-Tau

monitored by ThT fluorescence did not change significantly. During these aggregation assays, all parameters, including buffer pH, ionic strength, inducer type and concentration (in case of K18-Tau), incubation temperature, and shaking speed, were kept as explained in the main text. In summary, these results revealed no significant effect of the presence of AN-SP or taBODIPY dyes on the aggregation kinetics of Aβ peptide and Tau protein as monitored by ThT fluorescence.

Supplementary Note 2:

In order to explore possible variations between different preparations of Aβ peptide batches, we performed the experiments shown in **Figure S6.** The Aβ(1-42) sample that we used here was different from the one used in the main text as it was purchased from AnaSpec (Cat. No. AS-20276) and pretreated using the following procedure[1]: Peptides were dissolved in a 10% ammonium hydroxide solution in water at 0.5 mg/ml. The solution was incubated for 10 min at room temperature and then bath sonicated for 5 min. The samples were aliquoted, flash frozen in liquid nitrogen, lyophilized, and stored at −80°C.

Figure S6 shows that the Aβ(1-42) sample from this preparation led to higher initial ThT fluorescence at the start of the aggregation process compared to the result shown in **Figure 2** in the main text. This result suggests that this Aβ preparation contained some Aβ aggregates from the very beginning. Consequently, the difference in the fluorescence increases between AN-SP and ThT was not as pronounced as in the experiments shown in **Figure 2** in the main text. However, consistent with the results in **Figure 2** in the main text, also in this Aβ(1-42) preparation, AN-SP fluorescence increased faster during the first three hours of aggregation than ThT fluorescence (**see Figure S6D**). The taBODIPY, due to its large uncertainty after normalization, makes drawing a conclusion from this batch of Aβ difficult.

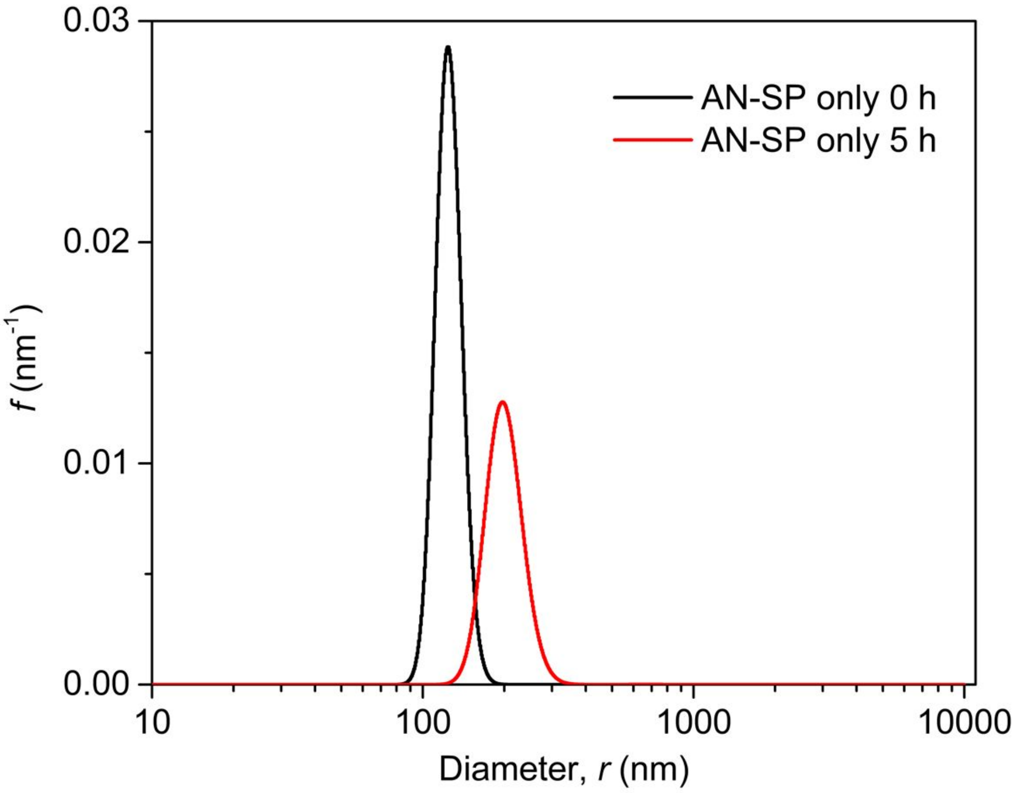

Figure S1. Particle size distribution of AN-SP in Aβ(1-42) aggregation. Estimated number-based distribution of the hydrodynamic radii of the colloidal particles. The particle size and relative polydispersity (STD/Mean) increased from (126 nm / 11 %) to (207 nm / 19%) during the initial 5 h of incubation at 37 °C with 300 rpm shaking speed. The buffer's contribution to light scattering was less than 1%, and therefore was negligible. Aβ aggregation buffer solution contains 100 mM NaCl, 10 mM Tris-HCl with pH 7.4, 5% (v/v) DMSO.

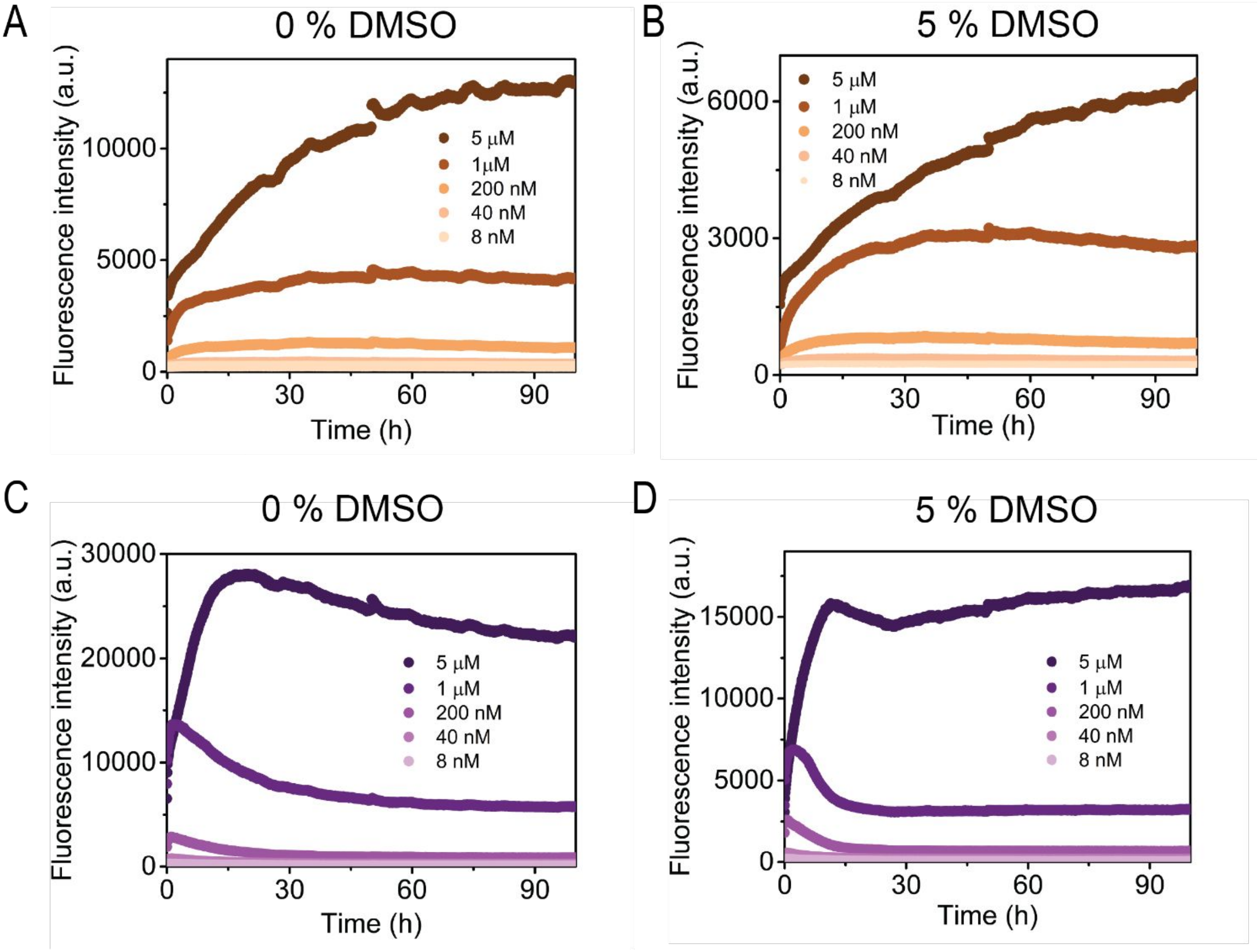

Figure S2. Control experiments of monitoring fluorescence intensity of various concentrations of AN-SP or taBODIPY in the absence of amyloids and in the presence of 0 % or 5 % DMSO in the solution. A) Fluorescence intensity of various concentration of AN-SP in aqueous solution with 0 % DMSO as a function of time. **B)** Fluorescence intensity of various concentration of AN-SP in aqueous solution with 5 % DMSO as a function of time. **C)** Fluorescence intensity of various concentration of taBODIPY in aqueous solution with 0 % DMSO as a function of time. **D)** Fluorescence intensity of various concentration of taBODIPY in aqueous solution with 5 % DMSO as a function of time. The data points shown as solid circles are the average of at least three measurements.

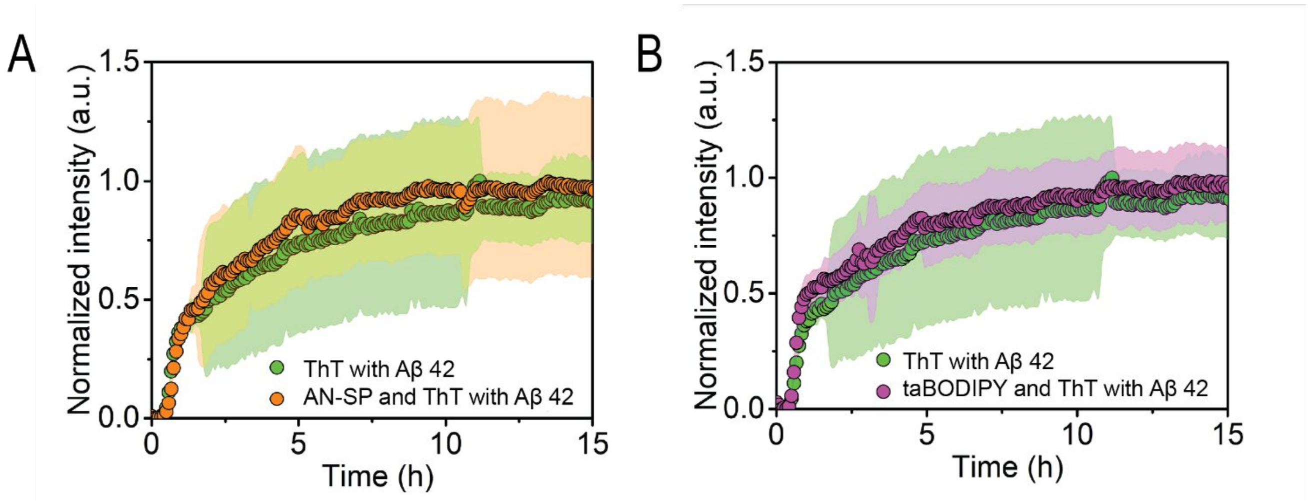

Figure S3. Control experiment to test a possible effect of AN-SP or taBODIPY dye on the aggregation kinetics of Aβ(1-42) peptide. A) ThT fluorescence over time (Ex: 440 ± 20 nm / Em: 490 ± 20 nm) in the absence (green) and presence (orange) of 12.5 μM AN-SP dye. **B)** ThT fluorescence over time in the absence (green) and presence (pink) of 12.5 μM taBODIPY dye. The data points shown as filled circles are the average of at least three measurements. The shaded region shows the standard deviation.

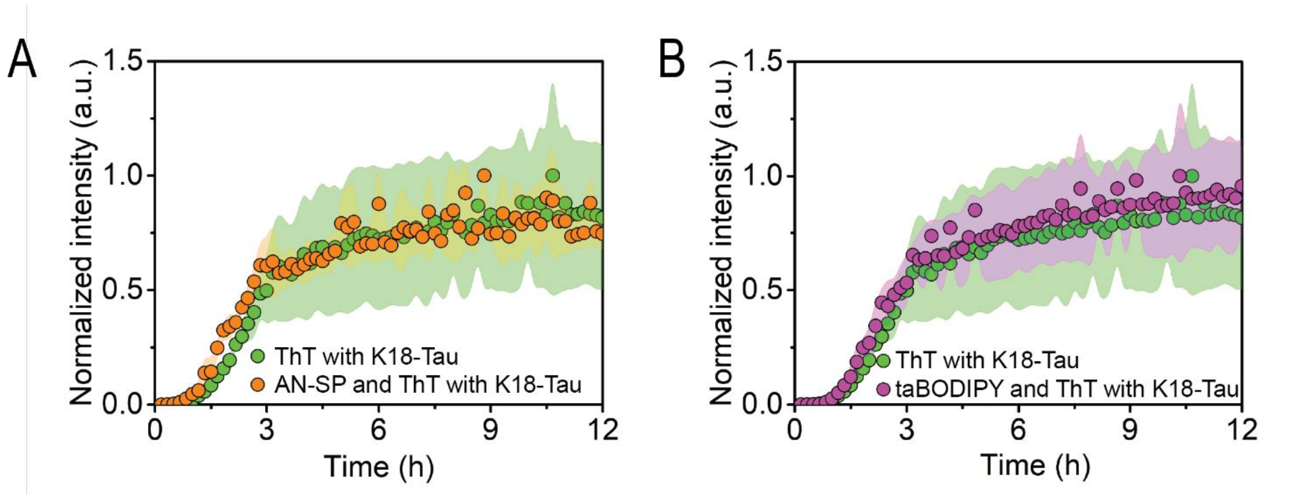

Figure S4. Control experiment to test a possible effect of AN-SP or taBODIPY dye on the aggregation kinetics of K18-Tau protein. A) ThT fluorescence over time (Ex: 440 ± 20 nm / Em: 490 ± 20 nm) in the absence (green) and presence (orange) of 12.5 μM AN-SP dye. **B)** ThT fluorescence over time in the absence (green) and presence (pink) of 12.5 μM taBODIPY dye (pink). The data points shown as

filled circles are the average of at least three measurements. The shaded region shows the standard deviation.

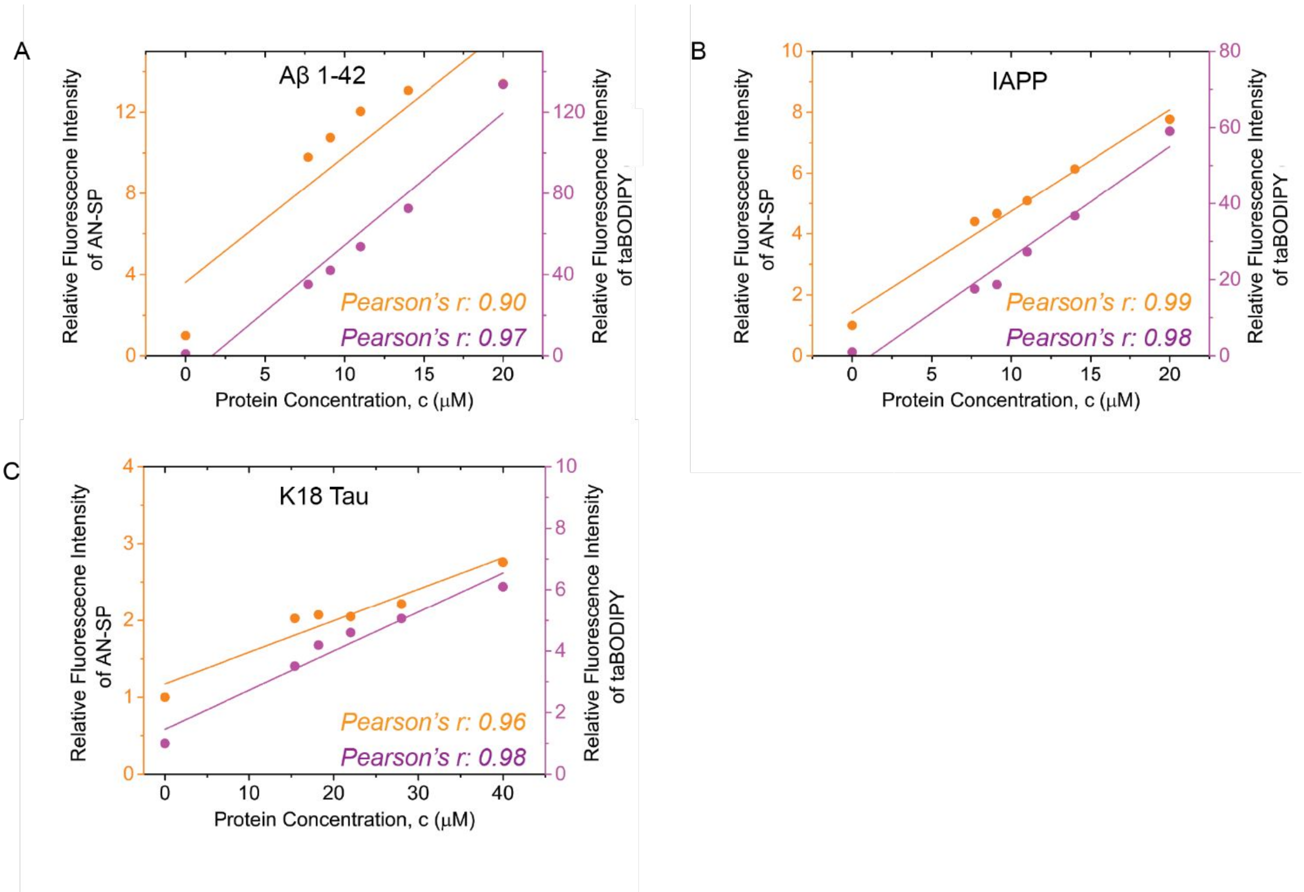

Figure S5. Relative fluorescence intensity of AN-SP or taBODIPY as a function of the concentration of aggregates of Aβ, IAPP or Tau. A) Relative fluorescence intensity of AN-SP (orange) or taBODIPY (purple) in the presence of different concentrations of aggregated Aβ(1-42) and a linear least squares regression fit to the data. **B)** Relative fluorescence intensity of AN-SP (orange) or taBODIPY (purple) in the presence of different concentrations of aggregated IAPP. **C)** Relative fluorescence intensity of AN-SP (orange) or taBODIPY (purple) in the presence of different concentrations of aggregated K18-Tau. To form aggregates, the Aβ(1-42) , IAPP and Tau samples were incubated at 37 °C for 20 h with 300 rpm shaking speed. The concentration of AN-SP was 4 μM the one of taBODIPY was 1 μM.

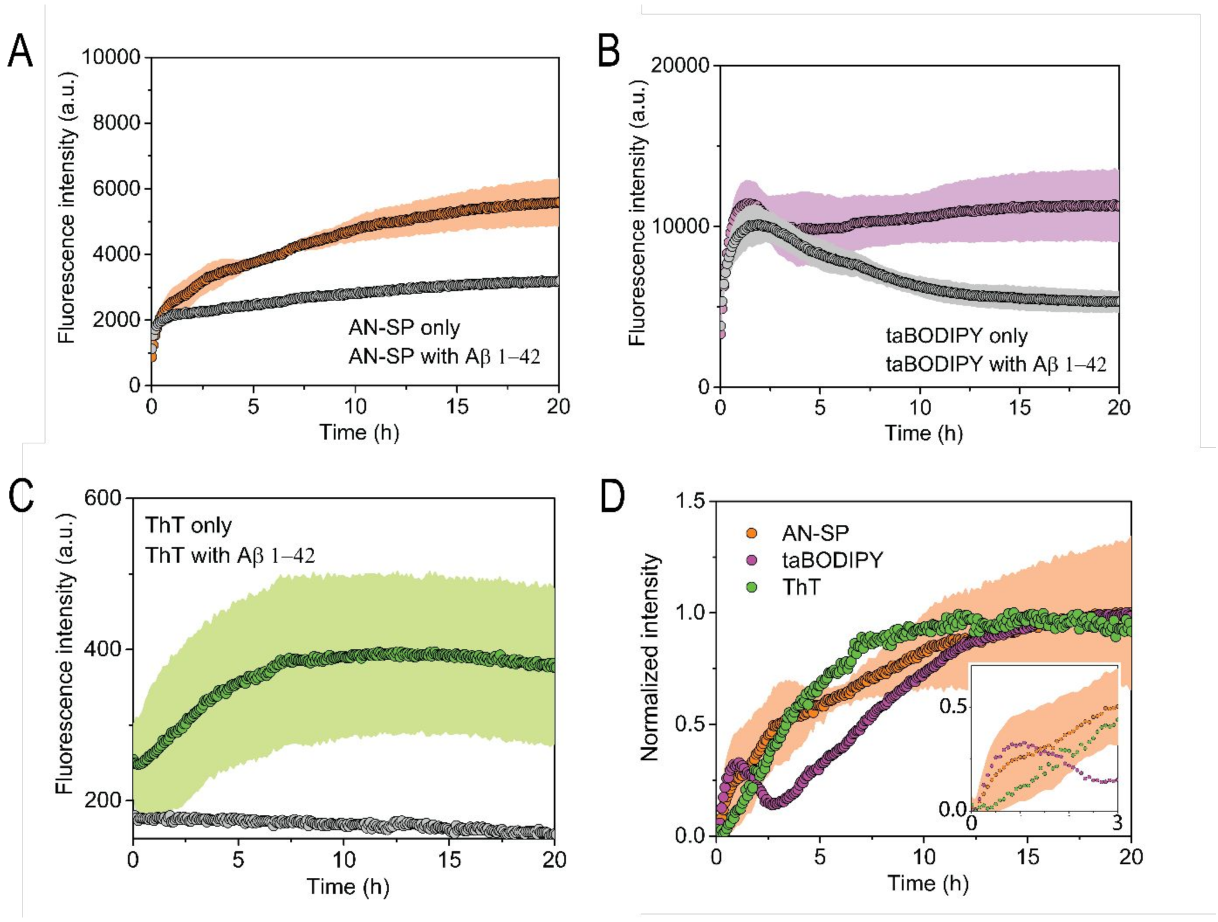

Figure S6. Aggregation kinetics of Aβ(1-42) peptide using an Aβ(1-42) batch different from the one used for the results presented in the main text. A) Aggregation of Aβ(1-42) monitored using AN-SP (orange) and blank control of AN-SP in the absence of Aβ(1-42) (grey). **B)** Aggregation of Aβ(1-42) monitored using taBODIPY (pink) and blank control of taBODIPY in the absence of Aβ(1-42) (grey). **C)** Aggregation of Aβ(1-42) monitored using ThT (green) and blank control of ThT in the absence of Aβ(1-42) (grey). **D)** Normalized aggregation curve after subtracting the blank control for AN-SP, taBODIPY, and ThT. The Inset shows the normalized aggregation curve monitored using AN-SP, taBODIPY, and ThT during the first three hours of aggregation. Each data point shown is the average of at least three measurements, except for the ThT data, which represents the average of two measurements. In all four panels, the shaded region shows the standard deviation for each data set. The regions shaded in grey for the blank controls are small and sometimes not visible. In panel D, the data for ThT and taBODIPY do not show the shaded region because of the large error from propagation of uncertainty. (see **Supplementary Note 2** for details)

Table S1. Half-time $t_{1/2}$ and lag-time t_{lag} of Aβ(1-42) aggregation using ThT assays in the presence or absence of different concentrations taBODIPY or AN-SP. The p values result from a statistical comparison of $t_{1/2}$ or t_{lag} values of aggregation in the presence of only ThT with the $t_{1/2}$ or t_{lag} values from aggregation in the presence of ThT combined with either taBODIPY or AN-SP at the indicated concentrations.

	ThT (10 μM)	ThT + various conc. of taBODIPY (μM)			ThT + various conc. of AN-SP (μM)		
		1	5	12.5	1	5	12.5
$t_{1/2}$ (min)	94.3±9.6	98.8±39.6	80.0±18.2	100±56.9	97.8±21.1	72.3±34.9	106.3±19.8
p value		0.99	0.94	0.99	0.99	0.97	0.96
t_{lag} *(min)*	54.3±5.0	58.8±27.6	37.7±13.7	60.0±40.9	57.8±14.7	32.3±26.9	66.3±13.8
p value		0.99	0.88	0.99	0.97	0.96	0.91

* Unpaired two-sample *t*-test revealed that the $t_{1/2}$ and t_{lag} values of Aβ(1-42) aggregation are not significantly different (all p values are >> 0.05) between conditions where only ThT is present or ThT is present together with either taBODIPY or AN-SP.

Table S2. **Half-time $t_{1/2}$ and lag-time t_{lag} of K18-Tau aggregation using ThT assays in the presence or absence of different concentrations taBODIPY or AN-SP.** The p values result from a statistical comparison of $t_{1/2}$ or t_{lag} values of aggregation in the presence of only ThT with the $t_{1/2}$ or t_{lag} values from aggregation in the presence of ThT combined with either taBODIPY or AN-SP at the indicated concentrations.

	ThT	ThT + various conc. of taBODIPY (μM)			ThT + various conc. of AN-SP (μM)		
	(10 μM)	1	5	12.5	1	5	12.5
$t_{1/2}$ (min)	153.2±3.1	131.9±6.5	121.8±8.2	145.2±4.1	147.7±4.0	132.7±4.3	128.7±4.4
p value		0.44	0.45	0.53	0.64	0.14	0.13
t_{lag} *(min)*	75.2±8.2	53.4±9.5	42.6±7.8	63.2±11.1	38.1±11.0	62.4±11.7	53.3±11.9
p value		0.78	0.58	0.89	0.67	0.88	0.82

* Unpaired two-sample *t*-test revealed the $t_{1/2}$ and t_{lag} of K18-Tau aggregation are not significantly different (all p values are >> 0.05) between conditions where only ThT is present or ThT is present with either taBODIPY or AN-SP.

Supplementary References

1. Ryan, T. M., Caine, J., Mertens, H. D., Kirby, N., Nigro, J., Breheney, K., Waddington, L. J., Streltsov, V. A., Curtain, C., Masters, C. L., & Roberts, B. R. Ammonium hydroxide treatment of Aβ produces an aggregate free solution suitable for biophysical and cell culture characterization. *PeerJ* **2013**, 1, e73.
